# Tool Condition Monitoring of the Cutting Capability of a Turning Tool Based on Thermography

**DOI:** 10.3390/s21196687

**Published:** 2021-10-08

**Authors:** Nika Brili, Mirko Ficko, Simon Klančnik

**Affiliations:** Faculty of Mechanical Engineering, University of Maribor, Smetanova ul. 17, 2000 Maribor, Slovenia; mirko.ficko@um.si (M.F.); simon.klancnik@um.si (S.K.)

**Keywords:** Tool Condition Monitoring, turning, Convolutional Neural Network, Deep Learning, Industry 4.0, thermography, tool wear, Inception V3

## Abstract

In turning, the wear control of a cutting tool benefits product quality enhancement, tool-related costs‘ optimisation, and assists in avoiding undesired events. In small series and individual production, the machine operator is the one who determines when to change a cutting tool, based upon their experience. Bad decisions can often lead to greater costs, production downtime, and scrap. In this paper, a Tool Condition Monitoring (TCM) system is presented that automatically classifies tool wear of turning tools into four classes (no, low, medium, high wear). A cutting tool was monitored with infrared (IR) camera immediately after the cut and in the following 60 s. The Convolutional Neural Network Inception V3 was used to analyse and classify the thermographic images, which were divided into different groups depending on the time of acquisition. Based on classification result, one gets information about the cutting capability of the tool for further machining. The proposed model, combining Infrared Thermography, Computer Vision, and Deep Learning, proved to be a suitable method with results of more than 96% accuracy. The most appropriate time of image acquisition is 6–12 s after the cut is finished. While existing temperature based TCM systems focus on measuring a cutting tool absolute temperature, the proposed system analyses a temperature distribution (relative temperatures) on the whole image based on image features.

## 1. Introduction

The evolution of the machining industry is based on automation, modern smart systems, unmanned machining, dynamic autonomous control of processes, etc. One of the keystones of this transition is the digitalisation of manufacturing, which is called Industry 4.0—the fourth industrial revolution [1]. Despite all the technological innovations, many decisions in individual or small series production are still made by humans. One example is the assessment of tool wear and the decision to change the cutting tool, which is usually left to the machine operator. There is no easy way for a machine operator to determine tool wear. One needs to rely on experience and visual inspection of the cutting edge of the cutting tool at the end of the cut. One also monitors the sound of the process (vibrations), the roughness of the workpiece surface, the colour (temperature) of the chips, the dimensional accuracy of the workpiece, etc.

Researchers are often confronted with the question of how to determine tool wear with the TCM System (Tool Condition Monitoring System). By introducing a reliable smart system that makes decisions instead of humans, it is possible to eliminate any error in judgement that may occur due to the human factor (e.g., inexperience). Several advantages can be obtained by monitoring the wear of a cutting tool. The condition of the cutting tool is one of the main factors affecting the quality of the final product [2] therefore the tool wear prediction is critical for product quality control [3]. However, not only the quality of the product is affected by the wear of the cutting tool. The financial aspect is also needs to be taken into account. Optimal costs can be achieved only if the cutting tool is changed at the right time—just before the quality of the machined surface does not meet the requirements due to the worn cutting tool. Replacing precipitately is related directly to the higher cost of a new cutting tool and additional changeover time. The use of a worn cutting tool has a negative effect on both the quality of the product and the machine tool load. TCM systems are crucial for cost-optimised manufacturing and play a fundamental role in Industry 4.0 [4] in the contest of error minimization and productivity maximization [5]. Since the cutting tool is clamped and heats up considerably during turning, various indirect systems without contact with the cutting insert are suitable methods for tool wear monitoring. Different sensors can be used, for example, sensors for measuring cutting forces, acoustic emission, vibration, temperature, motor current/power, sound, surface roughness. On the other hand, there are various possibilities for the tool wear dimensional measurement, such as Imaging Technology, which, however, usually lead to machine downtimes and, thus, to longer manufacturing times [6]. It is possible to determine the tool wear of the cutting tool during turning using a neural network, where the inputs may be; for example, cutting speed, feed rate, depth of cut and rake angle [7]; cutting speed, feed rate, depth of cut, and three force components [8]; cutting speed, feed rate, time, the width of cut, and the force ratio of the cutting force to the thrust force [9]; cutting force, acoustic emission, and vibration sensor signals [10].

The main focus was on studies that involve the analysis of temperatures during machining. What has already been researched: correlations between cutting speed, cutting temperatures, and consequent secondary hardening during hard turning of T15 HSS (High Speed Steel) [11]; determination of the optimal cutting parameters considering minimum flank wear and tool temperatures for turning a silicon carbide particulate reinforced Al 7075 matrix composite [12]; the effect of vibration and cutting zone temperature on surface roughness and tool wear [13]; a mathematical model of a milling tool’s flank wear based on the acquisition of the real-time cutting force and temperature [14]. Another approach is the Machine Vision method, which analyses the image of the cutting tool during turning [15] or face milling process [16]. Tool breakage detection can be done using Deep Learning methods that analyse the characteristics of the current [17] or forces [18] of a milling machine spindle, or forces during turning. Automatic prediction of the remaining life of a cutting edge is possible using image recognition with the special software Neural Wear [19]. A useful study for TCM systems investigated the relationships between machining parameters (cutting speed, feed rate, depth of cut), response parameters (flank wear and surface roughness), and sensorial data (cutting force, vibrations, acoustic emissions, temperature, and motor current). Using the statistical model ANOVA (analysis of variance) and the RSM (Response Surface Methodology)-based optimisation approach, it was shown that the cutting force is the most reliable and current is the less reliable sensor data. The temperature and the cutting speed are by far the most related parameters [20]. A similar approach, based on ANOVA, has been used to analyse the correlations between cutting parameters, surface roughness [21], and various vibrations [22]. An automated burr and slot measurement was developed on the basis of computer vision software and scanning electron microscope images [23].

Unlike other authors who typically classify cutting tool wear according to the degree of wear and the location where the wear occurs, Mamledesai et al. [24] introduced the TCM, which classifies cutting tool wear according to a tool change policy. The system detects whether a tool produces a conforming or non-conforming part. The method used is based on Machine Vision, Convolutional Neural Network (CNN), and Transfer Learning (TL). Brili et al. [25] were able to use a thermal IR (infrared) camera to monitor the cutting process successfully. Furthermore, thermographic images of chips were analysed with the CNN, a well-known Machine Vision model. This system sorts tools into 3 classes according to the wear level: no wear, low wear, and high wear. The cutting tool wear is determined during a turning process, which is achieved by mounting an IR camera to the revolver and taking images. The said images do not exhibit the cutting tool, because it is being obstructed by the emerging chips. 

The literature review demonstrates the usage of various approaches different from TCM. In the recent review paper on indirect Tool Condition Monitoring systems [4], neither thermography nor the IR camera are mentioned. In this work, a completely new approach for TCM for a turning tool is presented: A system that determines whether the cutting tool is suitable for further machining or not, based on the thermographic image of the cutting tool after the cut has been finished. The thermographic image made with the IR camera combines two approaches: Computer Vision and temperature measurements. Thermographic images were taken immediately after the end of the cut and then in the next 60 s. A Deep Learning method, more precisely a Convolutional Neural Network Inception V3, is used to analyse the images and classify them into 4 different classes: no, low, medium, and high tool wear. 

The existing temperature based TCM systems have focused on the absolute cutting tool temperature measurement, the proposed system based on the cutting tool temperature distribution (relative temperatures) is diverse. The whole thermographic image is an input to the Neural Network and the tool wear is categorised based on the features of the image. Furthermore, a use of Deep Learning for the analysis of thermographic images in the field of TCM is a novelty compared to existing research. By using feature-based method analysing relative temperature differences instead of absolute temperature measurement, the temperature dependent TCM system becomes more universal and robust. 

This paper is structured as follows: Section 2 presents the experimental setup and the CNN network used in this work; Section 3 reports the Results and Discussion, which describes the conclusions of the work; Section 4, Future work, presents topics that will be studied in the future.

## 2. Materials and Methods

The work presented in this paper has two main objectives: (I) To prove that thermographic images of cutting tools after the turning process are suitable for determining tool wear, and (II) to find out when is the best time to take cutting tool thermographic images with the IR camera.

The cutting tool has the highest temperatures and the largest temperature gradients immediately after the turning process. Over time, the temperature differences disappear. The idea of the work is not to measure the absolute temperatures of the cutting tool, but the whole thermographic image, especially the temperature differences are observed at different areas of the cutting tool. Therefore, it is important to verify how long after turning it is possible to control the tool wear with the IR camera.

The work presented in this paper was divided into the following steps: (I) Experiment: Acquisition of thermographic images of the cutting tools (Big Data processing); (II) Training a CNN model: Thermographic images were divided into different groups (depending on the time of acquisition), and a Convolutional Neural Network was trained on each group; (III) Testing the model: The trained model was tested on unknown images; (IV) Determination of the optimal time for image acquisition.

### 2.1. Experiment

The turning process is affected by various factors, which are a consequence of machine properties, tools, mount type, cutting parameters, and external disturbances. Some of the mentioned factors can be corrected, while others are unsusceptible to any control efforts to set them straight. 

The input parameters were held constant during the execution of the experiment. The control parameter was varied (cutting tool wear). Thermographic images of the cutting tool were monitored for each wear level. Every tool insert made 13–15 cuts. The number of cuts was determined and conditioned with getting the maximum quantity of images made by an individual insert. Simultaneously, it was assured that any significant change in the insert wear level during each image series was avoided. 

### 2.2. Categorising Tool Wear Levels

Practically wise, the result is paramount—successful machining with the equipment and product in mind. Although scientists often turn their focus to the cutting tool wear type and its causes, wear levels were inspected, and, consequently, the cutting tool’s adequacy for further machining was estimated. The main aim was to develop an intelligent system that determines the state of the cutting tool automatically based on its wear level (no, low, medium, and high wear). The system’s mission is the recognition of a worn insert which is not suitable for further machining, where the type of wear is not essential. 

It is imperative to think about another aspect—the difference between coarse and fine machining. The former happens at the first stage of cutting, where the majority of the material gets removed. Here, the surface finish is not the main concern, nor is the dimensional precision. Fine machining, on the other hand, is usually performed as the last machining operation (where additional operations, e.g., grinding or honing are not required), after which the final surface finish is achieved. 

The same insert wear criteria do not apply to both coarse and fine machining. An insert with low wear can still be adequate for coarse machining, but is not suitable where strict dimensional tolerances and low roughness are required. Table 1 depicts the appropriate tool for each machining type, depending on the tool wear. 

The wear of inserts was determined before the experiment was executed, i.e., based on two methods: Empirically (an expert’s diagnosis backed by their knowledge and experience) and Niakiev’s method [26] (indirect tool wear estimation by measuring the workpiece’s diameter). Both methods are discussed in detail in the paper [25]. Criteria for workpiece’s diameter deviation by Niaki’s method (Table 1) was determined regarding to the dimensional requirements, the diameter of the workpiece, and the type of cutting insert, considering that toolmaking is characterized by narrow tolerances. Workpiece’s diameter was measured after each cut at three different locations of the workpiece using a micrometer. The mean value of the three measurements was calculated. An expert’s assessment was determined before each cut.

### 2.3. Wear Monitoring and Image Acquisition Time

The state of a cutting tool was recorded with an IR camera, which, alongside the standard image output, also captures temperatures on the object’s surface. The camera that has been used was a Flir (Flir Systems, Inc., Wilsonville, OR, USA) model E5, with the following specifications: resolution 120 × 90 pixels, maximum image capture rate 9 Hz. This camera is considered to be a low-tier model. Although faster cameras with higher resolution are also available, it was found that the mentioned model already satisfies the given requirements. Its resolution is adequate for discerning tool wear based on thermographic images. The mid-range computer (Asus with AMD Ryzen CPU and 8 GB of RAM) took seven seconds to process one image. The aforementioned frame rate of 9 frames per second was more than enough because of the relatively long image processing time, as well as the nature of the observed phenomena. One of the research objectives was picking the most suitable equipment possible, which was sufficiently good to fulfill its purpose. Implementing such a system in the industry, it is desirable for it to be economically feasible so that investment in the monitoring system pays off in less than a year. 

During the turning process chips obstruct the cutting tool. It is known that a chip absorbs the most heat generated by cutting [27]. It consumes approximately 70% of the heat with a cutting speed of 100 m/min. Residual heat dissipates to the tool (20%) and the workpiece (10%) [28]. Due to the high-temperature deviations during the turning (especially prominent at the first few cuts), it is sensible to set the IR camera’s colour scale according to the temperature of the heated object shown on the screen. Consequently, a thermographic image of the turning process portrays the temperature distribution better in the chips. Peripheral objects in the image are displayed in a uniform colour. The second limitation for efficient tool state monitoring is that chips obstruct the cutting knife. The task of the research was to confirm the possibility of making predictions about the tool (the insert) based on thermographic images. Therefore, image acquisition is possible only after the turning is complete, where there is no more contact between the tool and the workpiece.

Now a question arises: at which point in time after the turning is the temperature distribution distinguishable enough to infer about the cutting tool wear?

**Figure 1:**
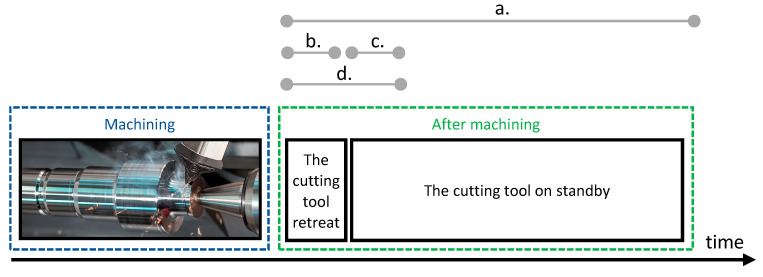
Thermographic image datasets, according to the time of their acquisition.

The image database has been divided into several smaller sets, depending on their acquisition time (Figure 1). Later, images were classified using the CNN taken from the following datasets: (a.) all images—60 s, (b.) 0–6 s after machining, (c.) 6–12 s after machining, (d.) 0–12 s after machining.

The cutting insert starts to cool right after the turning. The temperature gradient evens out proportionally with the time since turning up to the point when the image is captured. It was concluded that significant representations from the image get lost after a certain amount of time has passed. This is the reason why all the images were tested after turning (at most 60 s since the end of machining), as well as the initial few images after each cycle. 

### 2.4. Experimental Setup

The machining parameters were set before the beginning of the experiment (Table 2). These were held constant, i.e., parameters did not change during the model training. The model of intelligent cutting tool wear monitoring works by learning decision-making at constant conditions. The only input variable is the wear degree of the used cutting tool. 

**Figure 2:**
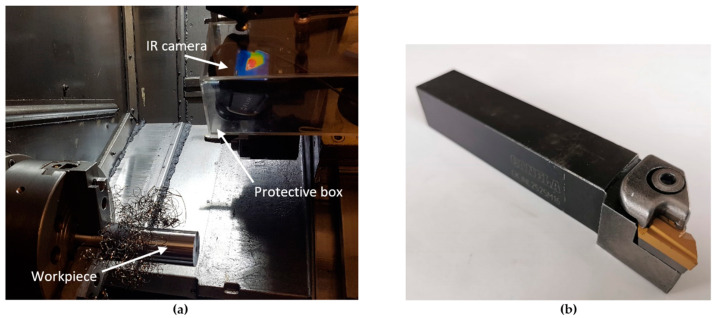
Experimental equipment: (**a**) Workpiece and IR camera, mounted closely against the cutting tool. (**b**) The cutting tool.

**Figure 3:**
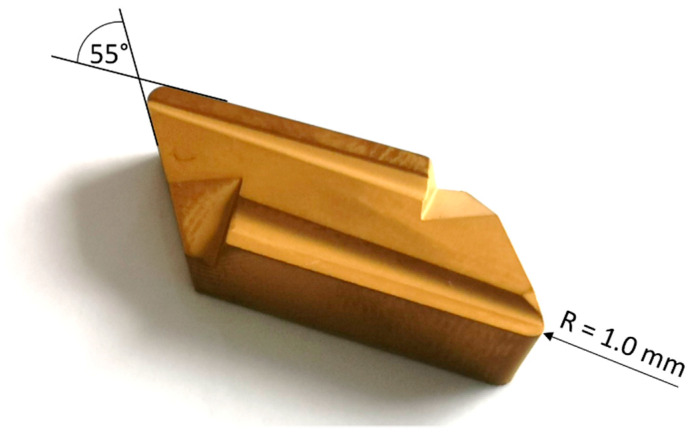
Cutting tool KNUX 160410L11.

Workpiece material chemistry, equipment used in the experiment and the cutting tool geometry are presented in the Table 3, Figure 2 and Figure 3, respectively. 

### 2.5. Convolutional Neural Network

Thermal images were classified using a Deep Learning method, more specifically: a Convolutional Neural Network. Why is a Convolutional Neural Network better for image recognition than a Classical Neural Network? The input to a Neural Network is a Table, where each row represents one input data with multiple parameters/variables (columns). Typically, 3 to 8 input variables are used [29], for example: number of revolutions, machining time, and cutting force [30]; material of the tool, the sharpening mode, the nominal diameter, the number of revolutions, the feed rate and the drilling length [31], depth of cut, cutting speed, and feed to the tooth [32]. A study using 20 input variables to predict tool life has shown that slightly more inputs are also possible, even if Fully Connected Neural Networks are used [19].

In the case of image recognition, each pixel represents one input variable for a greyscale image, and three input variables for a colour image in RGB (Red Green Blue). Thus, a colour image with a resolution of 120 × 90 pixels is an input file (one row in a Table) with 32,400 variables. A database consists of 9153 thermal images. Using a Classical Neural Network the input would be a Table of size 32,400 × 9153, which is too much data for a network with fully connected layers. However, the image could be used as an input, but, in this case, the features would have to be defined (with a feature extraction) that would represent the input to the Neural Network. So, personal perception would affect the efficiency of the system. Convolutional layers can reduce the input dimension. Based on the feature extraction method, convolutional neural networks find the best local features and focus on them. That is why CNN can compute such a large input. 

Since the development of a CNN is a very complex process, the Transfer Learning method is often used in Deep Learning. Meaning, a Neural Network is used, with an already optimised structure and filters. Only the last few layers of the CNN need to be unaltered on specific images. Different categories of images were defined, and the CNN learned on the learning database which images belonged to each class.

**Figure 4:**
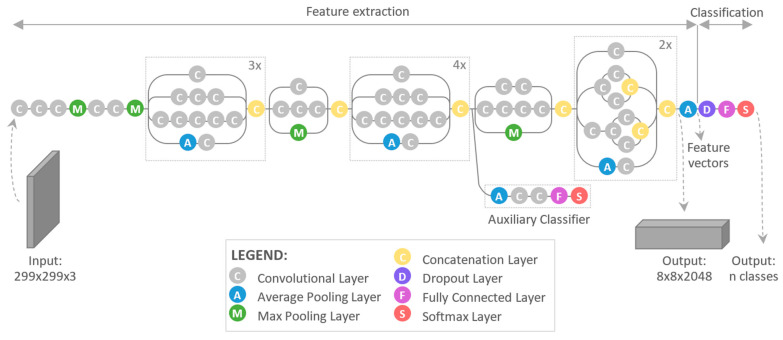
Architecture of Inception V3 [25].

The type of Convolutional Neural Network used in this study was Inception V3. The optimal architecture (Figure 4) and values of the Inception V3 filters were developed over several years by researchers Szegedy and others [33]. It is freely available, and can be found at www.tensorflow.org (accessed on 10 September 2019) under the Tutorials, Images, Image Classification tab.

## 3. Results and Discussion

In Section 2.2., how the thermographic images were sorted into distinct sets according to the time they were taken is presented. The results of each classification are presented in the form of a confusion matrix. The results of classifications are compared by the parameter Accuracy, however, Recall and Precision values were also taken into consideration [25].

Initially, the CNN was trained on images from the “training” set, and later the network was tested on the “test” set—images that the Neural Network had not seen yet. The sizes of training and test sets are given in Table 4. 

### 3.1. Complete Image Set (60 s)

Firstly, all of thermographic images were classified. The results are depicted in the confusion matrix in Table 5.

**Figure 5:**
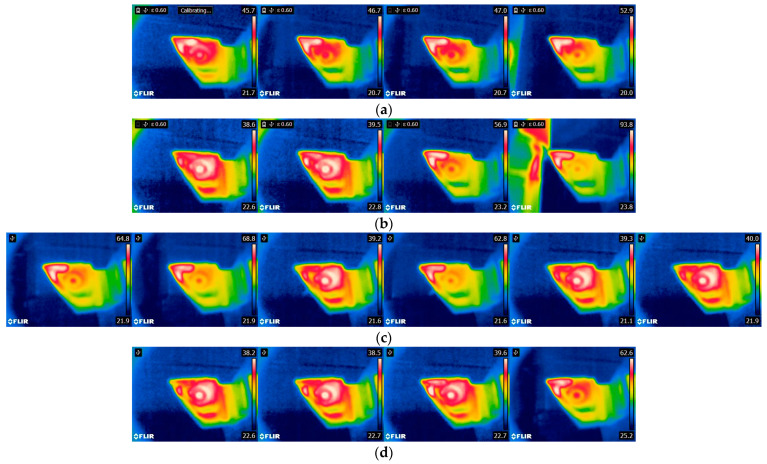
(**a**) “No wear”, but classified as “medium wear” (images belong to the turning series 9, 11, and 12—only images taken from the last series of turning were classified falsely, because the insert was not completely new); (**b**) “Medium wear”, but classified as “no wear” (images belong to the series 1 and 5—especially the first series of turning with medium insert wear, when it was not yet heated); (**c**) “Medium wear”, but classified as “high wear” (images belong to the series 9, 10, and 11); (**d**) “High wear”, but classified as “Medium wear” (images belong to the series 2 and 12).

After a brisk inspection of Table 5, it is evident that the CNN managed to classify 862 images correctly out of the total 880 images. Subsequently, the analysis of 18 false predictions was performed (Figure 5).

After inspection of the false predictions it was concluded that the CNN classified images from early—initial turning series into a lower wear severity class than it should. On the other hand, it classified images from later series (11th, 12th, etc.) into a wear level category that was too high. Abnormalities during a visual inspection were not observed. 

Additional probabilistic analysis for four images of an insert with a “high wear” that were classified as “medium wear” was performed, which was derived from the Softmax layer (Table 6). The analysis showed similar probabilities between the actual and false classes. Furthermore, the Neural Network’s second most probable guess always contained the correct class for each image. 

### 3.2. Other Classifications

Here, confusion matrices for the remaining categories are presented as follows: 0–6 s after machining (Table 7), 6–12 s after machining (Table 8), 0–12 s after machining (Table 9).

**Figure 6:**
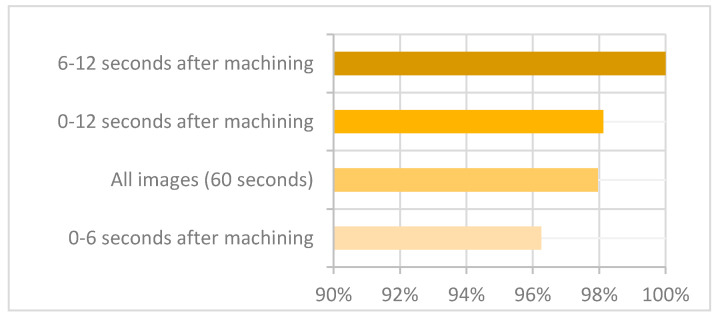
Accuracy for different classifications.

The performance of the model is represented most intuitively by accuracy [34], so all four classifications were compared by this evaluation indicator (Figure 6).

Figure 6 shows the accuracies of classifications for each dataset. The exceptional 100% accuracy was achieved for images in the category “6–12 s after machining”. That includes the moment when a cutting tool just leaves the workpiece, with the temperature gradient on the insert still clearly visible.

The Neural Network reached highly accurate predictions on the other datasets as well, namely 96.25% or more.

One crucial piece of information is hidden in the fact that the CNN did not make any predictions at the extremes of a diagonal in the confusion matrix. The model managed to avoid classifications of the “no wear” inserts into “high wear” class, and vice-versa. This information is very significant from a practical point-of-view, because such an error could represent high risk for damage and waste in the work process. It is assumed that, with an expansion of the training dataset, it is possible to enhance the classification’s accuracy additionally.

The analysis of the false classifications points to the incorrect predictions for at most two subsequent images. Most often, there was only one. If the algorithm was set in a way that it analyses, e.g., three subsequent images and determines the wear based on three identical classifications in a row, all false decisions could have been eliminated.

### 3.3. Discussion

The proposed study addresses the question of whether the condition of the cutting tool can be monitored using an IR camera. Thermographic images are analysed using Deep Learning, more specifically a Convolutional Neural Network. A turning tool was monitored for 60 s after the cut finished. The images were divided into different groups depending on the time of acquisition. The conclusions are as follows;

- The cutting tools were successfully divided into groups according to their suitability for further machining, also taking into account the type of machining (fine/rough).
- The proposed method was confirmed to be suitable for the TCM system.
- The tool can be monitored immediately after cutting or a few seconds later (e.g., when the tool turret is in home position).
- The accuracy for classifying the thermographic images ranged from 96.25% (0–6 s after machining) to 100% (6–12 s after machining).

The results are more than encouraging compared to similar studies: 85% accuracy for 37 tools that were divided into two groups GO or NO GO, according to the conformity of the part produced [24]; 5%, 10.7%, and 22% errors for estimated tool wear for milling tools [35]; 80% and 93% accuracy for tool breakage prediction using the Backpropagation Neural Network and CNN, respectively [17]; 6.7% absolute mean relative error between image processing systems based on an Artificial Neural Network and the commonly used optically measured VB index (Flank wear) [36]. Proposed smart system based on CNN and thermographic images is more accurate than other similar TCM systems.

The presented smart system for Tool Condition Monitoring and defining the suitability of the turning tool for further machining has shown high potential for industrial use due to its high accuracy, relatively low investment cost, and direct applicability of the decisions made. In line with the idea of Industry 4.0, decisions are shifted from humans to intelligent systems.

## 4. Future Work

The intelligent model for cutting tool wear and defect monitoring determines the tool state based on the thermographic image. The information crucial for the end-user (the machine operator) is whether the tool in question is still adequate for the type of intended machining or if it needs to be replaced. In the future, the aim is to develop a system that would serve recommendations to the operator about the cutting tool change. An algorithm will be constructed which will reduce the possibility of random false classifications of the cutting tool wear. 

Additional experiments will enable the expansion of the training dataset. The trained model will be tested on a new workpiece material and at altered machining parameters (cutting speed, feed rate, cutting depth, cutting tool). The research will be extended to the use of cooling/lubrication fluids, which is commonly used in industry. This way, the robustness of the trained model on unfamiliar conditions can be studied.

## Figures and Tables

**Table 1:** Classes of tool wear depending on the type of turning and according to workpiece’s diameter deviation (Nikaki’s method [26]).

	Insert Suitable for		Workpiece’s Diameter Deviation
	Rough Turning	Fine Turning	
**No wear**	YES	YES	Δ*D* < 0.02 mm
**Low wear**	YES	YES/NO ^1^	0.02 mm ≤ Δ*D* < 0.04 mm
**Medium wear**	YES	NO	0.04 mm ≤ Δ*D* < 0.07 mm
**High wear**	NO	NO	0.08 mm ≤ Δ*D*

^1^ Depending on the product requirements.

**Table 2:** Experimental Setup.

Experimental Setup	Parameter	Basic Experiment
Cutting parameters	Cutting speed	100 m/min
	Feed rate	0.2 m/min
	Cutting depth	0.25 mm
	Cooling	without
Workpiece material	Material Type	Alloy Steel
	Producer	SIJ Metal Ravne (Slovenia)
	Mat. No.	1.7225
	Hardness Rockwell C	27.8 HRC
	EN	42CrMo4
	Yield strength	≥900 N/mm^2^
Cutting tool	Type	KNUX 160410L11
	Producer	Sanstone (Zhuzhou Yifeng Tools Co., Ltd., Hunan, China)
	Material	Carbide insert with CVD coating
	Shape and Insert Included Angle	Parallelogram 55°
	Geometry	Negative
	Corner Radius	1.0 mm
	Cutting Edge Length	16 mm
	Relief angle	0°
Lathe	Producer	Okuma (Okuma Corporation, Aichi, Japan)
	Type	CNC (Computer Numerical Control) Lathe LC 30

**Table 3:** Chemical compositions (in weight %) of the workpiece material (according to the manufacturer’s specifications; SIJ Metal Ravne, Slovenia).

	Chemical Composition %							
Mat. No.	C	Si	Mn	Cr	Mo	Ni	V	W
1.7225	0.41	0.20	0.75	1.05	0.23	-	-	-

**Table 4:** Number of thermographic images for training and testing.

	Training Set	Test Set
All images (60 s)	8273	880
0–6 s after machining	920	80
6–12 s after machining	920	80
0–12 s after machining	1840	160

**Table 5:** Confusion matrix for the tool wear classification for all images.

		ACTUAL					
		No Wear	Low Wear	Medium Wear	High Wear	Sum	Precision
**PREDICTED**	No wear	216	0	4	0	220	98.2%
	Low wear	0	220	0	0	220	100%
	Medium wear	4	0	210	4	218	96.3%
	High wear	0	0	6	216	222	97.3%
	Sum	220	220	220	220	Accuracy: 97.95%	
	Recall	98.2%	100%	95.5%	98.2%		

**Table 6:** Probabilities of high wear insert images to be of some class.

	Image 1	Image 2	Image 3	Image 4
Medium wear	0.51866305	0.6532518	0.5108754	0.5324518
High wear	0.47833866	0.33923936	0.480641	0.44897473
Low wear	0.002657221	0.0071650357	0.008182266	0.018294167
No wear	0.000341077	0.000343807	0.0003013296	0.00027929

**Table 7:** Confusion matrix for the tool wear classification for images taken 0–6 s after machining.

		ACTUAL					
		No Wear	Low Wear	Medium Wear	High Wear	Sum	Precision
**PREDICTED**	No wear	19	0	1	0	20	95.0%
	Low wear	0	20	0	0	20	100%
	Medium wear	1	0	19	1	21	90.5%
	High wear	0	0	0	19	19	100%
	Sum	20	20	20	20	Accuracy: 96.25%	
	Recall	95.0%	100%	95.0%	95.0%		

**Table 8:** Confusion matrix for the tool wear classification for images taken 6–12 s after machining.

		ACTUAL					
		No Wear	Low Wear	Medium Wear	High Wear	Sum	Precision
**PREDICTED**	No wear	20	0	0	0	20	100%
	Low wear	0	20	0	0	20	100%
	Medium wear	0	0	20	0	20	100%
	High wear	0	0	0	20	20	100%
	Sum	20	20	20	20	Accuracy: 100%	
	Recall	100%	100%	100%	100%		

**Table 9:** Confusion matrix for the tool wear classification for images taken 0–12 s after machining.

		ACTUAL					
		No Wear	Low Wear	Medium Wear	High Wear	Sum	Precision
**PREDICTED**	No wear	39	0	1	0	40	97.5%
	Low wear	0	40	0	0	40	100%
	Medium wear	1	0	39	1	41	95.1%
	High wear	0	0	0	39	39	100%
	Sum	40	40	40	40	Accuracy: 98.16%	
	Recall	97.5%	100%	97.5%	97.5%		

## Abbreviations

The following physical quantities and abbreviations are used in this manuscript:

CNC	Computer Numerical Control
CNN	Convolutional Neural Network
HSS	High Speed Steel
IR	Infrared
RSM	Response Surface Methodology
TCM	Tool Condition Monitoring
*VB*	Flank wear
Δ*D*	Diameter deviation
